# Live synthesis of selective carbon dots as fluorescent probes for cobalt determination in water with an automatic microanalyzer

**DOI:** 10.1007/s00604-023-05975-w

**Published:** 2023-09-19

**Authors:** Alex Pascual-Esco, Pere Lleonart, Antonio Calvo-López, Julián Alonso-Chamarro, Mar Puyol

**Affiliations:** Group of Sensors and Biosensors, Department of Chemistry, Faculty of Sciences, Universitat Autònoma de Barcelona, Carrer dels Til·lers s/n, Bellaterra, 08193 Cerdanyola del Vallès, Spain

**Keywords:** Microreactor, Carbon dots, Fluorescence quenching, Microfluidics, Cobalt determination

## Abstract

A new strategy integrating the straight synthesis of carbon dots (CDs) and their direct use for the determination of heavy metals by means of fluorescence quenching is presented. The proposal consists of a modular analyzer, which includes a low temperature co-fired ceramics (LTCC) microreactor for the synthesis of CDs and a cyclic olefin copolymer (COC) microfluidic platform, which automatically performs a reverse flow injection analysis (rFIA) protocol for the determination of heavy metal ions in water by CD fluorescence quenching. As a proof of concept, nitrogen-doped CDs were synthesized from acrylic acid and ethylenediamine (ED) with quantum yields (QYs) of up to 44%, which are selective to cobalt. With the described system, we synthesized homogeneous CDs without the need for further purification and with the minimum consumption of reagents, and optimized fluorescence measurements can be performed with freshly obtained luminescent nanomaterials that have not undergone decomposition processes. They have an average hydrodynamic diameter of 4.2 ± 0.9 nm and maximum excitation and emission wavelengths at 358 nm and 452 nm, respectively. The system allows the automatic dilution and buffering of the synthesized CDs and the sample prior to the determination of cobalt. The concentration of cobalt was determined with good sensitivity and a limit of detection of 7 μg·L^−1^ with a linear range of 0.02–1 mg·L^−1^ of Co^2+^. Spiked tap water and river water samples were analyzed, obtaining recovery from 98 to 104%. This demonstrates the potential of the equipment as an efficient on-site control system for heavy metal monitoring in water.

**Graphical abstract:**

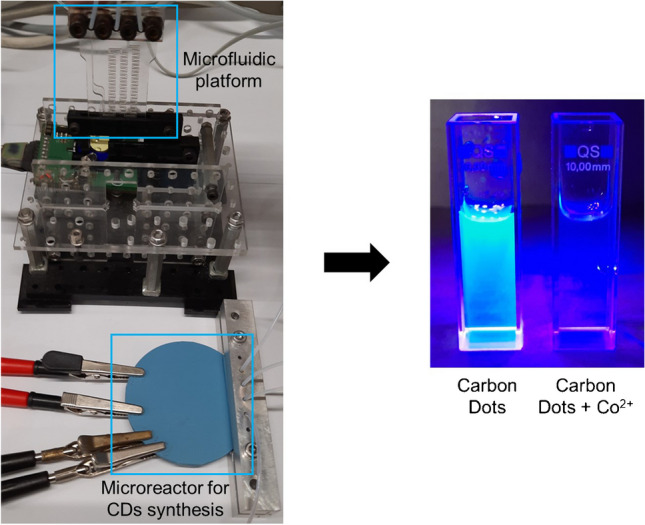

## Introduction

Environmental pollution is receiving more attention in the last decades. One of the most critical polluting agents are heavy metals, considering that they are not biodegradable and tend to accumulate in the ecosystem and different tissues of living beings [1]. Different heavy metals can be found naturally in the environment, but the rise of some human activities like mining, manufacturing, smelting, and the improper disposal of industrial wastes is contributing to an increase in their negative environmental and health impact [2–5]. The most common techniques used to analyze heavy metals include inductively coupled plasma mass spectrometry (ICP-MS), inductively coupled plasma optical emission spectrometry (ICP-OES), and atomic absorption spectrometry (AAS) [6, 7]. These techniques offer high analytical features in terms of sensitivity, accuracy, and precision but show some disadvantages for on-site monitoring because of the complexity and dimensions of the instrumentation, which hinders the possibility of carrying out measurements outside a laboratory and the need for specialized personnel [4]. In this sense, the miniaturization of analytical procedures through microfluidic devices solves some of the mentioned problems, increasing the portability and enabling the on-site continuous monitoring of a wide variety of analytes, including heavy metals [5, 8, 9].

Currently, with the fast expansion of nanotechnology, different photoluminescent nanoparticles have been investigated due to their potential applicability in sensing [10] and bioimaging [11], to list just a few fields. Their use as fluorescent probes for heavy metals analysis has proven to lead to better sensitivity and selectivity, improved detection limits, and faster response time [3, 12]. One type of photoluminescent nanoparticles that have recently drawn great attention from the scientific community are carbon dots (CDs). They are carbon nanoparticles with sizes below 10 nm [13] and have different characteristics to highlight as optical probes like their distinctive photoluminescence (strong fluorescence with tunable emission) [14] and good biocompatibility [15]. Compared to other luminescent molecules, CDs do not suffer from photobleaching, which is one of the major drawbacks of the formers, and compared to other luminescent nanomaterials such as quantum dots (QDs), CDs are water soluble directly from a one-pot synthesis [13], so it is not necessary to proceed with solubilization steps before its use. Due to their carbonaceous nature, they present lower toxicity than QDs [16, 17], which is in accordance with green chemistry. Finally, CDs have been successfully used to selectively detect different heavy metal ions in water by easy methods of preparation and functionalization [18, 19]. CDs can be easily modified by element doping [1]. More specifically, nitrogen doping originates an increase in fluorescence intensity of CDs, caused by changes in the electronic structure, and also leads to the formation of active sites [2, 20], which are useful for further surface functionalization in order to provide them with selectivity. However, the main bottom-up synthetic methods of CDs (hydrothermal synthesis and microwave-assisted synthesis) [1, 21] lack the required reproducibility to produce materials for analytical applications [21], which will directly impact the reliability of the final analytical result.

In order to face this issue, microreactor technology arises for synthesis process intensification [22–24]. It allows for enhancing reagents mixing and heat and mass transfer efficiency. In consequence, better control over the particle size distribution and a reduction in the consumption of energy and reagents are achieved. From the different substrate materials for microreactor fabrication, low temperature co-fired ceramics (LTCC) technology has some advantages like the capability to withstand harsh temperature and pressure conditions, chemical inertness, and compatibility with screen printing technology, which enables the easy integration of electrical components [11, 25, 26].

Taking this context into account, our proposal was the development of a modular automatic analyzer consisting of a cyclic olefin copolymer (COC) microfluidic system, which was optimized for the determination of heavy metals in water, and an LTCC microreactor for the direct synthesis of CDs and their use as fluorescent probes, without the need of any purification steps. The synthetic strategy used, an adaptation of a hydrothermal batch method, based on the use of a microreactor, takes profit of the advantages of a microfluidic continuous strategy that allows much better control of the chemical variables of the reaction (minimizing temperature gradients, increasing the speed of mixing, and controlling the reaction times), which improves the reproducibility of the synthesis processes. As an example of the integration of the optical probe synthesis and the water quality parameter analysis in a single unit, the microreactor was optimized to synthesize N-doped CDs, which were selective to cobalt(II), from two precursors, acrylic acid and ethylenediamine (ED) [27]. Therefore, the analytical microsystem was optimized to determine cobalt(II) in water samples, taking advantage of its fluorescence quenching effect on the CDs. Cobalt, which at trace levels is an essential element in the human body [28], is harmful at high concentrations, causing asthma, rhinitis, gastritis, and, in severe cases, cardiomyopathy [28, 29]. Although it is not a heavy metal that causes great concern, its determination in water served us perfectly as a model to validate our proposal.

## Experimental

### Reagents, materials, and preparation of precursors

All reagents, namely acrylic acid (99%), ethylenediamine (99%), citric acid (99%), sodium citrate tribasic (99%), quinine sulfate (90%), sulfuric acid (95%), and the metal salts Co(NO_3_)_2_ · 6H_2_O, Hg(NO_3_)_2_ · H_2_O, Pb(NO_3_)_2_, FeCl_3_ · 6H_2_O, Cu(NO_3_)_2_ · 3H_2_O, Ni(NO_3_)_2_ · 6H_2_O, Cd(NO_3_)_2_ · 4H_2_O, Zn(NO_3_)_2_ · 6H_2_O, NaNO_3_, CaCl_2_ · 2H_2_O, MgCl_2_ · 6H_2_O, and Cr(NO_3_)_3_ · 9H_2_O (> 98% for all the metal salts) were supplied from Merck Sigma-Aldrich (Barcelona, Spain) (https://www.sigmaaldrich.com/ES/en).

Solutions of the precursors for the CD synthesis and 0.1 M citric/citrate (pH 4) buffer were prepared in MilliQ water. The solutions used for the CD characterization, selectivity, and cobalt ion determination were prepared in the mentioned buffer.

For the different syntheses of N-doped CDs, 1.6 mL of acrylic acid (99%, density 1.05 g·mL^−1^) was dissolved in 10 mL of MilliQ water (2.33 M) and loaded in a glass syringe (Hamilton series Gastight 1000 TLL). Three different concentrations of the nitrogen source precursor were prepared: 0.5 mL, 1.0 mL, and 1.5 mL of ED (99%, density 0.9 g·mL^−1^) were dissolved in 20 mL of MilliQ water in each case (0.37, 0.75, and 1.12 M, respectively). For each synthesis process, two glass syringes were loaded with the same nitrogen source precursor.

LTCC 951 green tapes with various thicknesses were supplied by DuPont Corporation (Wilmington, DE, USA) (https://www.dupont.es/) and used to fabricate the microreactor: 254 μm thick DuPont 951PX green tapes and 114 μm thick DuPont 951PT green tapes. DuPont 5742 gold co-fireable conductor paste was used to print the gold resistor, and DuPont 6141 silver co-fireable paste was used to print the contact pads.

The microfluidic platform was fabricated with COC sheets of diverse thicknesses and grades, which were purchased from TOPAS Advanced Polymers GmbH (Florence, KY, USA) (https://www.topas.com/): 400 µm Topas 5013 COC and 25 µm Topas 8007 COC layers.

### Fabrication of the low temperature co-fired ceramics (LTCC) and cyclic olefin copolymer (COC) microfluidic platform

The LTCC microreactor was fabricated by a procedure previously developed by our research group [11]. Briefly, the different layers of the microreactor were designed with computer-aided design (CAD) software. The microfluidic channels and other elements were cut in the LTCC green tapes using a Nd:YAG Protolaser 200 (LPKF Laser and Electronics, Garbsen, Germany) (https://www.lpkf.com/en/). Then, the processed layers were aligned and thermolaminated with a hydraulic press (Talleres Francisco Camps, Granollers, Spain) (http://www.tallerescamp.com/). Fluidics comprises three inlets for the introduction of the synthesis precursors, a spiral-shaped microfluidic channel, and an outlet. The cross-section dimensions of the channel are 525 μm width and 290 μm height, while the total length is 630 mm. With this, the total volume of the microreactor channel is approximately 100 μL.

**Fig. 1:**
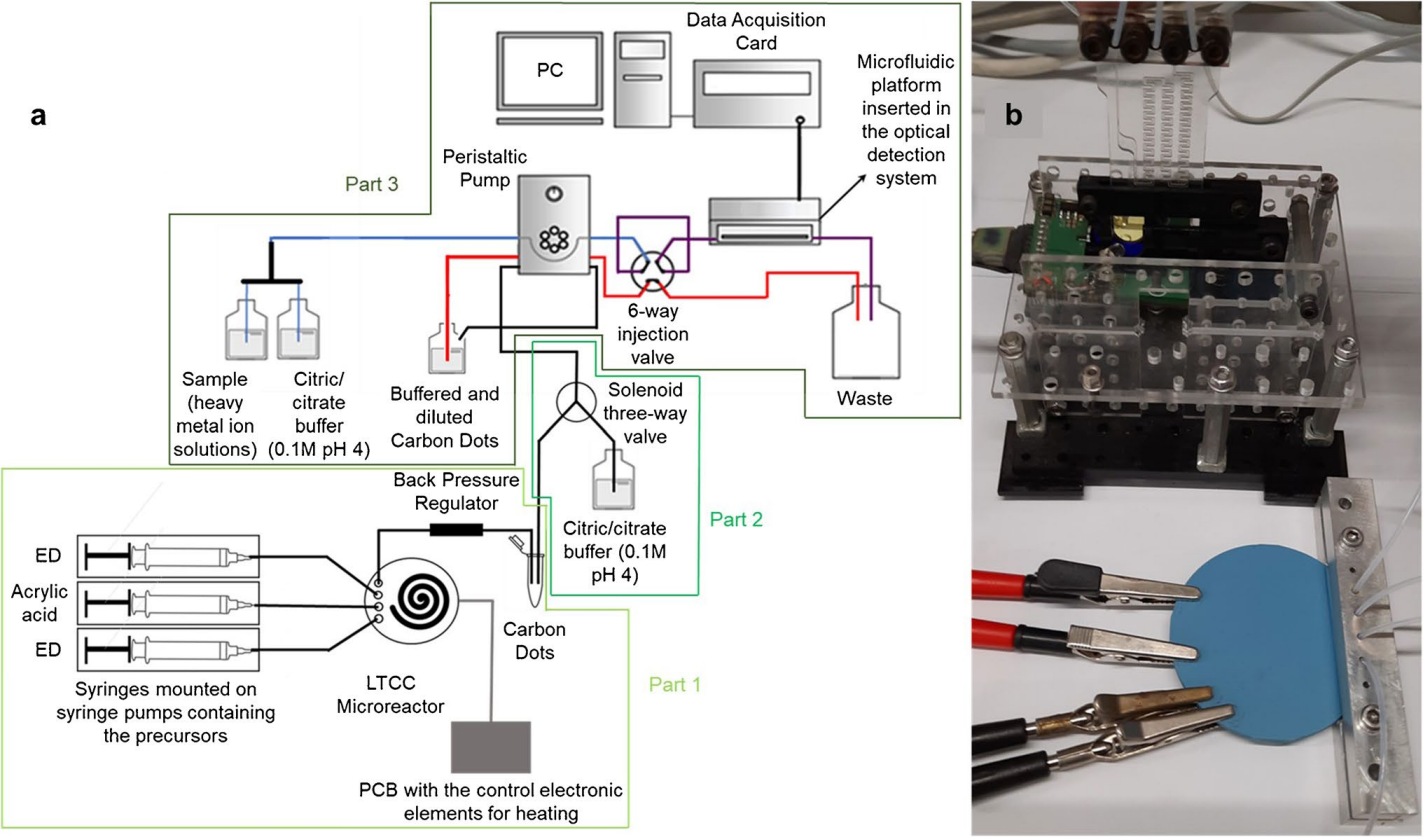
(**a**) Experimental setup used, including the synthesis of the CDs (part 1), their automated dilution (part 2), and the determination of Co^2+^ (part 3). The fluidic management equipment is also depicted. (**b**) Detailed image including the microreactor with fluidics (secured with a custom-built aluminum connector) and electrical connections, and the custom-made miniaturized optical detection system with an inserted microfluidic platform

The heating resistor, which occupies the same area as the microfluidic channel to reduce energy consumption, was screen-printed on the reverse of the fluidic inlets and outlet. Another LTCC green tape was laminated on top of the resistor, where the connecting pads were also screen-printed. The device with all the layers laminated was then sintered in a CBCWF11/23P16 programmable box furnace (Carbolite Gero, Hope Valley, England) (https://www.carbolite-gero.com/) following a two-step thermal profile, including a 1 h organic burnout at 350 °C and 1 h firing at 850 °C. Finally, a PT100 temperature sensor (Innovative Sensor Technology, Ebnat-Kappel, Switzerland) (https://www.ist-ag.com/en) was adhered to the microreactor using EPO-TEK H20E epoxy paste (Epoxy Technology, Billerica, MA, USA) (https://www.epotek.com/). The final device (Fig. 1b) has a thickness of 3 mm and a diameter of 6 cm.

The COC microfluidic platform that can be seen in Fig. 1b is 30 mm wide, 50 mm high, and 2 mm deep and has two inlets, a two-dimensional meander micromixer (0.8 mm wide and 1 mm deep), an optical flow cell (4.5 mm diameter and 1 mm deep), and an outlet. It was fabricated with a multilayered approach in the same way as the LTCC microreactor was. The process was also developed in our research group [8, 30], and the first step was the CAD design of the layers. Then, the different layers were micromachined on a Protomat S63 Computer Numerical Control (CNC) micromilling machine (LPKF Laser and Electronics Garbsen, Germany). As for the LTCC microreactor, when microfluidics were integrated into the COC substrate, the different layers were thermolaminated with the hydraulic press to obtain the final device.

### Carbon dots characterization and selectivity test

Absorption spectra of the synthesized CDs were registered with a UV-3101PC UV-Vis-NIR double beam spectrophotometer (Shimadzu, Kyoto, Japan) (https://www.shimadzu.com/), and fluorescence excitation and emission spectra were obtained with a Fluorolog FL3-11 spectrofluorometer (Horiba Jobin Yvon, Longjumeau, France) (https://www.horiba.com/int/scientific/). QY values were determined by applying the optically dilute measurement method [31] using quinine sulfate in 0.1 M sulfuric acid as a reference as follows:

$${\varphi }_{x}={\varphi }_{st}\times \frac{{I}_{x}}{{I}_{st}}\times \frac{{A}_{st}}{{A}_{x}}\times \frac{{\eta }_{x}^{2}}{{\eta }_{st}^{2}}$$

where *φ* is the photoluminescence QY, *I* is the integrated area of the corrected emission spectrum, *A* is the absorbance, and *η* is the refractive index of the solvent. The subindexes *x* and *st* refer to the sample and the reference standard, respectively. Considering that both the standard and sample were dissolved in water, the last term of the equation was ignored.

Fourier transform infrared (FTIR) spectra of the CDs were acquired with a Tensor 27 FTIR spectrophotometer (Bruker, Billerica, MA, USA) (https://www.bruker.com/en.html). High-resolution transmission electron microscopy (HR-TEM) images were collected using a Tecnai G2 F20 HR(S)TEM (Field Electron and Ion Company, Hillsboro, OR, USA) (https://www.fei.com), and dynamic light scattering (DLS) measurements were carried out in a Zetasizer Nano ZS (Malvern Panalytical, London, England) (https://www.malvernpanalytical.com/en) to check morphology and particle size distribution of the CDs.

To test the selectivity of the CDs, their emission spectra in the presence of different heavy metal ion solutions (Cd^2+^, Co^2+^, Cu^2+^, Cr^3+^, Fe^3+^, Hg^2+^, Ni^2+^, Pb^2+^, Zn^2+^) at a concentration of 10 mg·L^−1^ and other metal ions normally present in water (Na^+^, Mg^2+^, Ca^2+^) at a concentration of 1000 mg·L^−1^ were acquired per triplicate.

Emission spectra were recorded by mixing in a cuvette 0.5 mL of CD dispersion at the optimized dilution factor (to have an approximate absorption of 0.05 a.u. and avoid possible self-absorption effects) and 2.5 mL of citric/citrate buffer as reference emission value and with 2.5 mL of buffered solutions containing the mentioned metallic ions.

### Experimental setup

The complete system setup (Fig. 1a) is computer-controlled and consists of three main parts: (1) an automatic system for the synthesis of CDs (containing a fluidic management system and a temperature-controlled microreactor), (2) an interface (including the automated dilution of the CDs), and (3) the determination system of Co^2+^ (containing a fluidic management system and the COC microfluidic platform integrated into a miniaturized optical detection system).

Part 1): For the synthesis of the CDs, three syringes were filled with the precursors. The syringes were mounted on three NE-500 OEM syringe pumps (New Era Pump Systems Inc., Farmingdale, NY, USA) (https://www.newerainstruments.com/) and connected to the microreactor using 0.8 mm internal diameter Teflon tubing (Tecnyfluor, Barcelona, Spain) (https://www.tecnyfluor.com/). The connections were secured with FPM75 O-rings (Epidor, Barcelona, Spain) (https://epidor-srt.com/) and a custom-built aluminum connector. To improve mixing, acrylic acid (the carbon source) was introduced through the central inlet, while ED (the nitrogen source) was introduced through the other two inlets, as can be seen in Fig. 1a. The pressure inside the microreactor was regulated by a back-pressure regulator connected to the outlet. A pressure of 17 bars was applied to all the syntheses, and three different working temperatures were tested (150 °C, 170 °C, and 190 °C). The synthesis precursors were pumped at a flow rate of 3.33 μL·min^−1^ for each inlet (total flow rate of 10 μL·min^−1^), and considering that the internal volume of the microreactor is about 100 μL, the residence time of the reagents is of approximately 10 min. The temperature was controlled by means of a proportional-integral-derivative (PID) system implemented on a PIC18F4431 microcontroller (Microchip Technology Inc., Chandler, AZ, USA) (https://www.microchip.com/), receiving the sensor input [32].

Part 2): CDs from the synthesis were automatically diluted and buffered at pH 4 with the help of a 161T031 three-way solenoid valve (NResearch, West Caldwell, NJ, USA) (https://www.nresearch.com/). They were diluted 100 times with 0.1 M citric/citrate buffer; 0.8 mm internal diameter Teflon tubing was used for fluidic connections. A FlowTest automated controller (BioTray, Villeurbanne, France) (https://www.biotray.fr) controlled the operation of the solenoid valve. It was programmed through the dedicated CosDesigner software.

Part 3): For the determination of Co^2+^, a reverse flow injection analysis (rFIA) strategy was applied [30]. The diluted CDs were sequentially injected into the buffer (blank) and different Co^2+^-containing solutions. The microfluidic setup includes a pre-buffering step of samples that is automatically performed by using an in-line T connector mixer, through which the sample and the buffer are introduced in a 1:1 ratio. The flow management is performed with a Gilson Minipuls 2 peristaltic pump (Middleton, WI, USA) (https://es.gilson.com/), 0.8 mm internal diameter Teflon tubing, and 1.14 mm internal diameter Tygon tubing (Ismatec, Wertheim, Germany) (https://heidolph-instruments.com/es/start). An MVP six-port injection valve (Hamilton Company, Bonaduz, Switzerland) (https://www.hamiltoncompany.com/) was used to inject the CDs, and the connections with the microfluidic platform are secured with FPM75 O-rings. The microfluidic platform is introduced in a custom-made miniaturized optical detection system previously reported [8, 30]. Briefly, it contains an LED emitting at 365 nm, a band-pass filter, and a PIN photodetector integrated into a printed circuit board (PCB). The insertion of the platform is based on a “lock-and-key” concept [8] that allows a reproducible positioning of the device with respect to the LED and the photodetector. The signal is obtained with a data acquisition card. Some parameters affecting signal-to-noise ratio were previously optimized [30]. To summarize, for this specific work, the following conditions were applied: CD injection volume of 500 µL, flow rate of 1.5 mL·min^−1^, signal amplification of 10, and integration time of 0.1 s.

With this method, a continuous photoluminescent signal was established, with a maximum value when CDs were injected into a solution without cobalt, and quenched values when the solution contained cobalt. The fluorescence intensity, which is obtained as the peak height, was correlated with the Co^2+^ concentration using the Stern–Volmer equation [33].

### Analytical characterization for Co^2+^ determination in water

The relative fluorescence intensity can be plotted against the quencher concentration (in this case, cobalt), according to the Stern–Volmer equation.

$$\frac{{F}_{0}}{F}=1+{K}_{SV}\times [Q]$$

where *F*_0_ is the fluorescence intensity of the CDs in the absence of the quencher, *F* is the fluorescence intensity of the CDs in the presence of the quenching species Q (Co^2+^), and *K*_*SV*_ is the Stern–Volmer quenching constant, which indicates the sensitivity of the method. According to the literature, the CD quenching mechanism of Co^2+^ can be associated with static quenching [27].

Calibration plots were obtained by injecting per triplicate 100-time diluted CDs in standard solutions with different concentrations of cobalt, namely 0.01 mg·L^−1^, 0.05 mg·L^−1^, 0.1 mg·L^−1^, 0.5 mg·L^−1^, and 1 mg·L^−1^. This CD dilution ensures a good signal-to-noise ratio and avoids the inner filter effect (the absorbance is less than 0.04 at the maximum excitation wavelength of 365 nm). The repeatability of the measurement, calculated as relative standard deviation (RSD), was checked by performing ten injections of CDs into a solution containing 0.05 mg·L^−1^ of Co^2+^. The limit of detection (LOD) and limit of quantification (LOQ) were calculated as three times and ten times the standard deviation of the blank signal (citric/citrate buffer) divided by the slope of the calibration plot (K_SV_), respectively.

To assess the practical applicability of the synthesized CDs to determine Co^2+^, spiked tap water and river water (Besòs river, Spain) samples were evaluated. The spiking process was done to undiluted samples prior to their analysis, and the concentration range was chosen, taking into consideration the maximum admissible limit of cobalt in drinking water set at 0.1 mg·L^−1^ by the US EPA. Therefore, four spiked concentrations of Co^2+^ (0.10 mg·L^−1^, 0.25 mg·L^−1^, 0.50 mg·L^−1^, and 0.75 mg·L^−1^) were tested. Recovery rates and % RSD were calculated for three measurements, considering these concentrations as the true value because the concentration of Co^2+^ in tap and river water samples was under the LOD (1 µg·L^−1^) determined by ICP-OES.

## Results and discussion

### Synthesis of carbon dots optimization

The modification of the reaction conditions such as temperature, pressure, reaction time, and molar fraction of the precursors allows modulating QYs because different proportions of the two fractions of CDs (crystalline and amorphous) can be obtained, but these do not affect significantly the maximum excitation and emission wavelengths. The effect of the concentration of the nitrogen precursor and the temperature over the resulting CDs were evaluated by comparing the corresponding QYs; 17 bars were the pressure chosen for all the syntheses due to channel occlusion observed at lower pressures [11].

**Fig. 2:**
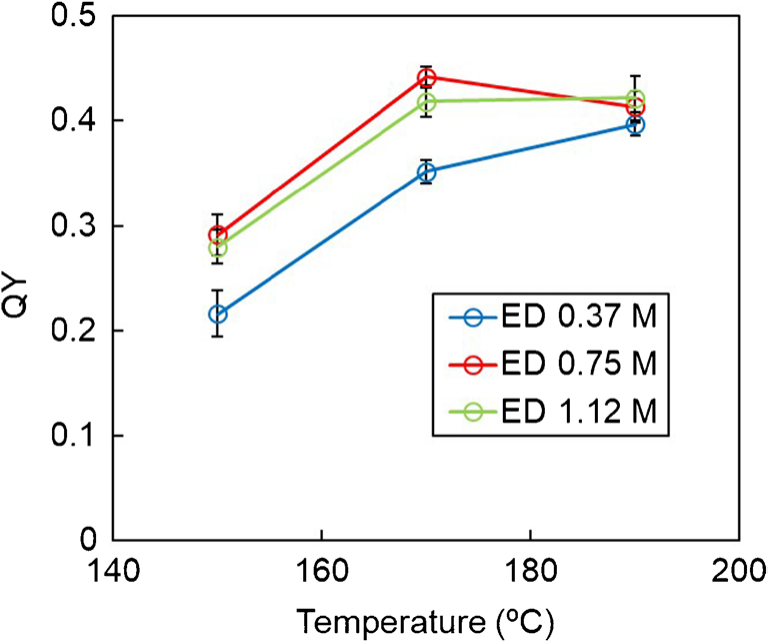
Comparison of the QYs of the CDs under different temperatures and different concentrations of nitrogen precursor (ED). All the syntheses were performed at 17 bars and with a solution of 2.33 M of carbon precursor (acrylic acid)

As can be seen in Fig. 2, the temperature of the synthesis had an important effect on the QY of the CDs obtained, showing a maximum at 170 °C. Performing the reaction at higher temperatures can increase the carbonization, obtaining a carbon core-based product, which is more photostable but has a lower QY [34]. Additionally, the effect of the precursor concentration was also noticeable. When the concentration of the ED solution was 0.37 M, lower QY values were obtained, indicating that the reaction was incomplete, while when the concentration of the ED solution was 1.12 M, the QY was lower than that obtained with the solution containing 0.75 M of ED, which means that an excess of ED in solution was affecting the final CDs QY.

Therefore, the optimal synthetic conditions to obtain the highest QY (44%) were using a solution containing 0.75 M of the nitrogen precursor and performing the synthesis at 170 °C and 17 bars.

### Characterization of carbon dots and selectivity tests

**Fig. 3:**
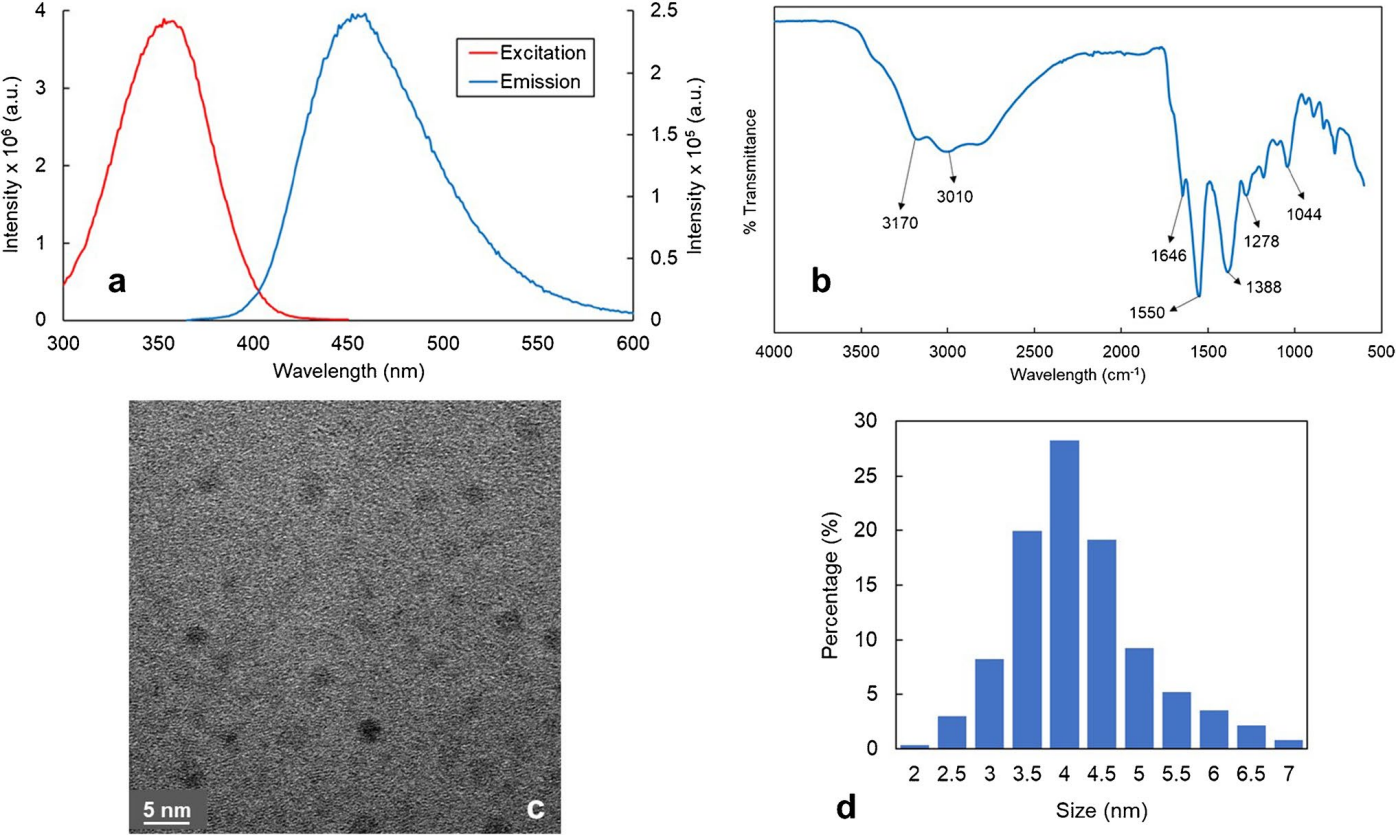
(**a**) Photoluminescence spectra of the synthesized CDs diluted 100 times to have an approximate absorption of 0.04 a.u; (**b**) FTIR spectrum of the undiluted CDs; (**c**) HR-TEM image of the undiluted CDs; and (**d**) DLS measurement of the undiluted CDs

To follow the optical performance of the synthesized CDs, fluorescence emission and excitation spectra were examined (Fig. 3a). CDs showed wide excitation and emission bands with maximum intensities at 358 nm and 452 nm, respectively, obtaining a Stokes shift of approximately 100 nm.

An FTIR spectrum (Fig. 3b) was taken to characterize the functional groups present on the surface of the CDs. The following peaks can be observed, which can be assigned to different functional groups: the broad peak in the 3500–2500 cm^−1^ area comes from stretching vibrations of O-H groups. Bands at 3170 cm^−1^ and 3010 cm^−1^ correspond with N-H of an amide group and with C-H stretching vibrations, respectively. The peak at 1646 cm^−1^ can be assigned to the stretching vibration of C = O, the peak at 1550 cm^−1^ to the C = C stretching vibration, the peak at 1388 cm^−1^ belongs to the bending vibration of N-H, the one at 1278 cm^−1^ is from the asymmetric stretch of C-N, and, finally, the peak at 1044 cm^−1^ is attributed to C-O aromatic stretching [35].

Results indicated that nitrogen functionalization was successful and that carboxyl, hydroxyl, amino, and amide bonds exist on the surface of the CDs, which can act as anchor sites to adsorb more Co^2+^ on the CDs’ surface to enhance the analysis sensitivity [27].

The morphology and size distribution of the CDs were studied through HR-TEM images and DLS measurements (Fig. 3c, d, respectively). Results show that the CDs have a quasi-spherical shape and an average hydrodynamic diameter of 4.2 ± 0.9 nm. However, it was very difficult to obtain high-quality contrast TEM images due to the formation of two fractions of CDs, one with a crystalline structure (carbon core particles) that can be identified thanks to the observation of the diffraction planes and another one that is amorphous and suggests the formation of polymer dots. These polymer dots have molecular fluorophore moieties embedded, causing the enhancement of the QY and making it more difficult to obtain TEM images [34]. Both fractions determine the optical characteristics of the final nanomaterial to be employed as an optical probe. In this sense, the use of the microreactor is of special importance because it allows better control of the synthetic conditions (temperature, pressure and precursors mixing) than batch methods. This assures to obtain not only more homogeneous CDs but also a high reproducibility between the different syntheses [36].

**Fig. 4:**
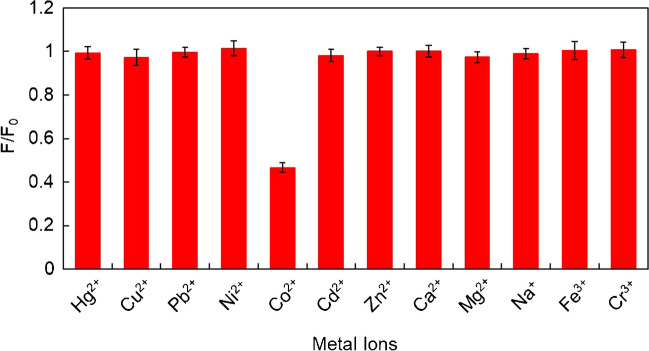
Representative study showing the selectivity by the representation of the ratio between the fluorescence after the addition of various metal ions to the CDs and the fluorescence of the CDs

Selectivity is an important issue to assess the application of synthesized CDs as fluorescent probes. To evaluate selectivity, emission spectra of the CDs in the presence of different metal ion solutions (Ca^2+^, Cd^2+^, Co^2+^, Cu^2+^, Cr^3+^, Fe^3+^, Hg^2+^, Mg^2+^, Na^+^, Ni^2+^, Pb^2+^, Zn^2+^) were recorded. As can be seen in Fig. 4, the fluorescence intensity of CDs was only quenched by Co^2+^ among all the other metal ions studied, suggesting that the synthesized CDs are selective to Co^2+^. The interactions between Co^2+^ ions and the functional groups of the surface of the CDs (-COOH, -OH, -CONH-) formed non-luminescent complexes, which can explain the observed decrease in fluorescence intensity [29, 33].

### Analytical performance

**Fig. 5:**
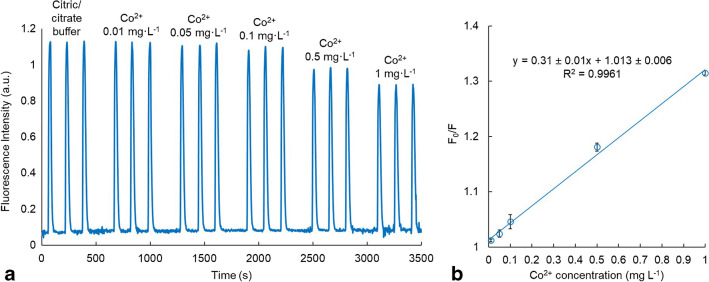
(**a**) Fluorescence intensity signal of the microsystem (obtained with an excitation source emitting at 365 nm, a CDs injection volume of 500 µL, and a flow rate of 1.5 mL·min^−1^), where different peaks appear when the CDs were injected into the buffer (intensity value in the absence of the quencher (*F*_0_)) and different standard solutions of the quencher (F). (**b**) Stern–Volmer plot of the quenching effect of Co^2+^ on the fluorescence of the CDs

Different concentrations of cobalt were used to evaluate the analytical performance of the system. Figure 5a shows the transient fluorescence intensity signal of the microsystem by injecting the 100-time diluted CD dispersions into 0.1 M citric/citrate buffer (blank) and solutions of different concentrations of Co^2+^. The obtained calibration plot (Fig. 5b) shows a good linear correlation (*R*^2^ = 0.996) in the range of 0.02 to 1 mg·L^−1^ of Co^2+^. The Stern–Volmer quenching constant, which is equivalent to the sensitivity, obtained is 0.31 ± 0.01 L·mg^−1^.

The repeatability of the system was calculated as the % RSD, obtaining a value of 1.3%, thus indicating good repeatability. Additionally, the LOD and LOQ were calculated, obtaining the values of 7 μg·L^−1^ and 21 μg·L^−1^ of Co^2+^, respectively. The maximum admissible limit of cobalt in drinking water is not mentioned by the World Health Organization (WHO) nor the European Union (EU). As a reference value, the United States Environmental Protection Agency (US EPA) has fixed the maximum admissible limit at 0.1 mg·L^−1^ [5].

The analytical features of other reported CD-based optical methods are summarized in Table 1. As it can be seen, these methods reach slightly lower LODs and wider linear ranges. However, the method presented in this work allows obtaining CDs with the highest QY, which ensures a strong signal even in case of possible reagent decomposition and as optical probes in the developed analyzer; they procure an LOD that is well below the reference value for Co^2+^ set by the US EPA for drinking water. The automated synthesis of the CDs in the LTCC microreactor and its direct use for the determination of Co^2+^ without any purification saves time and manual intervention. Compared to the reported manual methods based on batch fluorescence measurements, the proposed analytical method fulfills other analytical features such as automation and portability.

**Table 1:** An overview of recently reported CD-based optical methods for the determination of Co^2+^ ions

Precursors	QY (%)	LOD (µg·L^−1^)	Linear range (mg·L^−1^)	Reference
Pyridoxal 5-phosphate and ethanediamine	15	3	0–3.5	[15]
L-cysteine	27	2	0.06–2.9	[37]
p-phenylenediamine and asparagine	16	1	0.02–3.8	[7]
Carbopol 934 and diethylenetriamine	39	27	0–2.4	[29]
Citric acid and L-cysteine	-	5	0.005–5.9	[38]
Acrylic acid and ethylenediamine	23	15	0.06–3.5	[27]
Acrylic acid and ethylenediamine	44	7	0.02–1.0	This work

Regarding the Stern–Volmer quenching constant (*K*_*SV*_), it has been doubled (0.31 ± 0.01 L·mg^−1^) compared with the synthetic method in batch (0.14 L·mg^−1^) [27] indicating an improvement in the sensitivity.

Taking into consideration that the response time of the CDs-Co^2+^ interaction to generate the analytical signal is approximately 2 min, the overall sample throughput was calculated at 30 h^−1^. If, for monitoring purposes, an analyte was determined in the process solution to be monitored 4 times a day and the analyzer was calibrated with 5 standard solutions and the blank, all of them per triplicate, once a day, 30 measurements a day would be necessary to perform. This involves the synthesis of 150 µL of CDs a day (they are diluted 100 times in the system before the injection). In this situation, the microreactor would continuously perform the synthesis during 15 min. If other monitoring schedules were performed, the synthetic procedure would last differently.

The present modular prototype also has some limitations. To demonstrate its applicability for on-site monitoring, it would be necessary to compact the whole instrumentation to make it more portable, robust, automatic, and autonomous. Long-term studies of the performance in continuous operation of the system should be carried out to detect possible instrumental instability problems related to the fluidic microsystem or other interference effects derived from analyzing complex polluted sample matrices. Accuracy should also be assessed by comparison with a reference method for Co^2+^ determination in water samples.

### Real sample analysis

The high selectivity and sensitivity of the synthesized CDs toward Co^2+^ suggest that the current fluorescent sensor based on CDs can be applied for measuring Co^2+^ in real water samples. To confirm that, spiked tap water and river water samples were analyzed. The results obtained are summarized in Table 2. The calculated recoveries are in the range between 98 and 104%, and RSD for three measurements is lower than 4% in all cases. This validates the application of the proposal for online Co^2+^ determination in real water samples.

**Table 2:** Determination of Co^2+^ in spiked tap and river water samples (*n* = 3)

Sample	[Co^2+^] added (mg·L^−1^)	[Co^2+^] found (mg·L^−1^)	Recovery (%)	RSD (%)
Tap water	0.10	0.104 ± 0.007	104	2.8
	0.25	0.25 ± 0.02	101	2.5
	0.50	0.50 ± 0.02	100	1.6
	0.75	0.77 ± 0.05	102	2.5
River water	0.10	0.10 ± 0.01	102	3.8
	0.25	0.24 ± 0.02	98	2.9
	0.50	0.49 ± 0.02	99	2.0
	0.75	0.75 ± 0.04	100	2.0

## Conclusions

In this work, we describe a new analytical strategy for the direct use of freshly synthesized nanoparticles by microreactor technology as optical probes in automatic microanalyzers. The complete system is modular and consists of three main parts: a CD synthesis module with an LTCC microreactor, a dilution interface for the CDs obtained, and a fluorescence detection module containing a COC microsystem. All of this is computer-controlled for the automatic determination of heavy metals by fluorescence quenching. As a model system for the validation of the proposal, we synthesized highly efficient (high QYs) N-doped CDs from acrylic acid and ED to selectively determine Co^2+^ ions in water samples.

To demonstrate the adequate performance of the system, Co^2+^ was selectively detected among other heavy metal and metal ions usually present in water with high sensitivity and an LOD and LOQ of 7 µg·L^−1^ and 21 µg·L^−1^, respectively, that are well below the reference value set by the US EPA for drinking water. The target analyte was successfully determined in spiked tap water and river water samples with good accuracy and precision, thus demonstrating the viability to integrate the synthesis of the CDs prior to the analysis and their application as optical probes for Co^2+^ determination in water. This represents an advantage not only in terms of automation of the synthesis but also in terms of the use of a fresh optical material that has not undergone any degradation prior to analysis. The reduced dimensions of the microreactor and the microfluidic platform allow for minimizing the amounts of reagents, which would favor less maintenance in a real application for heavy metals monitoring.

The proposed system offers the possibility of expanding the number of analytes to determine by synthesizing other types of selective CDs by alternating other precursors (containing N, S, P, etc.) and the reaction conditions (molar ratios and temperatures).

Further miniaturization and integration of the different modular parts are currently being performed for fluidic management by using computer-controlled micropumps and microvalves to improve portability and robustness. To demonstrate the applicability of the equipment in real on-site monitoring and for validation purposes, it will be necessary to evaluate the effect of organic matter present in water samples in the fluorescence quenching of the CDs, to perform long-term studies, compare results with a reference method, and analyze certified reference materials.

## Data Availability

Data will be made available on request.
